# Accelerating the Original Profile Kernel

**DOI:** 10.1371/journal.pone.0068459

**Published:** 2013-06-18

**Authors:** Tobias Hamp, Tatyana Goldberg, Burkhard Rost

**Affiliations:** 1 Bioinformatics & Computational Biology - I12, Department of Informatics, Technical University of Munich, Garching/Munich, Germany; 2 Center of Doctoral Studies in Informatics and Its Applications (CeDoSIA), Technical University of Munich Graduate School, Garching/Munich, Germany; 3 Institute of Advanced Study (TUM-IAS), Garching/Munich, Germany; 4 New York Consortium on Membrane Protein Structure (NYCOMPS) and Department of Biochemistry and Molecular Biophysics, Columbia University, New York, New York, United States of America

## Abstract

One of the most accurate multi-class protein classification systems continues to be the profile-based SVM kernel introduced by the Leslie group. Unfortunately, its CPU requirements render it too slow for practical applications of large-scale classification tasks. Here, we introduce several software improvements that enable significant acceleration. Using various non-redundant data sets, we demonstrate that our new implementation reaches a maximal speed-up as high as 14-fold for calculating the same kernel matrix. Some predictions are over 200 times faster and render the kernel as possibly the top contender in a low ratio of speed/performance. Additionally, we explain how to parallelize various computations and provide an integrative program that reduces creating a production-quality classifier to a single program call. The new implementation is available as a Debian package under a free academic license and does not depend on commercial software. For non-Debian based distributions, the source package ships with a traditional Makefile-based installer. Download and installation instructions can be found at https://rostlab.org/owiki/index.php/Fast_Profile_Kernel. Bugs and other issues may be reported at https://rostlab.org/bugzilla3/enter_bug.cgi?product=fastprofkernel.

## Introduction

### Profile kernels provide state-of-the-art accuracy

The characterization of proteins often begins with their assignment to different classes. Examples for such classes are protein families, distant structural relations, or sub-cellular localization. GO, the Gene Ontology [1], is the most comprehensive functional vocabulary and defines over 38,000 different 'GO terms', i.e. classes into which a protein could be grouped. The simplest classification is through *homology-based inference* [2–4]. A PSI-BLAST [5] or HHBlits [6] query against a database with annotations such as Swiss-Prot [7] creates a list of proteins that have reliable experimental annotations and are sequentially similar to the target. Choosing the annotation of the best hit for the query then constitutes one simple means of annotating function [4,8].

Such a naive prediction method has disadvantages: query results are usually ordered by the e-value or the HVAL [2] of the best local alignment. This is not the best choice for all classification problems. A membrane-integral domain, for example, might be located at the N-terminus of the target, whereas the alignment with the best hit begins near the C-terminus. Therefore, advanced machine learning methods such as *Neural Networks* or Support Vector Machines (SVMs) often outperform simple homology-based inference [9–11], even for very small classes [12].

These methods represent proteins in a high-dimensional space, as given, for example, by the frequencies of the 20 amino acids in a protein. Some of the most popular and accurate classifiers are sequence-profile based kernels in conjunction with SVMs [13–19]. They do not require a protein to be represented explicitly, but only implicitly via dot-products to other proteins. Without this limitation, even the score of a local alignment can be turned into a kernel function and harness the advantages of the maximum-margin hyperplanes computed by SVMs [15–17].

### Methodological limitations difficult to address

This advantage, however, comes at a computational cost. The dot products required for training are stored as kernel matrices, which are quadratic in the number of training samples. Furthermore, in order to classify a new query, dot products have to be calculated with respect to all *Support Vectors*. Their number, however, is typically proportional to the amount of classes and template proteins. This puts strong limitations on data set sizes and some kernels that are sufficiently fast for today’s searches might become infeasible soon because the growth of the bio-sequence data far outpaces the growth of computing hardware.

Current solutions to the problem of data set sizes that are preventative for training include the use of linear SVMs, keeping only parts of the kernel matrix in memory or massive parallelization. All three options are mostly inapplicable to profile kernels. The first two (linear SVMs; caching the kernel matrix) are complicated because explicit sample vectors are either unknown or too large and calculating the same kernel values multiple times slows down training unacceptably. The second (parallelization) has, to the best of our knowledge, not been implemented by any state-of-the-art method, yet.

In some cases, predictions can be accelerated much more elegantly: if a kernel operates directly in the feature space, the normal vector separating one class from the other may be calculated explicitly, instead of implicitly via support vectors and associated weights. This reduces predicting a new query to calculating a single dot product.

### Accelerating the original profile kernel

Here, we show how to apply these concepts to the kernel introduced by the Leslie group [13]. It is arguably the most popular profile-based kernel today and its outstanding performance for many tasks has been repeatedly confirmed [16–19]. We have recently applied it in the development of a state-of-the-art method for the prediction of sub-cellular localization, LocTree2 [20]. On top of its high performance, the original profile kernel has other advantages, such as the ability to extract sequence motifs from trained SVMs. In particular, its hyper-planes can be made explicit as long as also the underlying k-mer trie based algorithm is modified accordingly.

Consequently, our first and most important improvement is calculating the matrix product of input profiles and pre-computed SVM normal vectors at full use of the k-mer trie based data structure. This corresponds to an efficient and highly parallel classification of many protein profiles with many SVMs at the same time, without the need for multiple CPU cores. Secondly, addressing the training phase, we can now distribute the computation of a single kernel matrix to an arbitrary amount of parallel processes. Due to optimizations of procedures required both for training and testing, also existing un-parallelized routines now run about five times faster than in the original implementation. Finally, we have combined all the necessary steps for training a classifier in a single program. It automatically calculates the kernel matrix, learns a user-defined SVM-based multi-class model, extracts and compresses the SVM normal vectors and stores everything as a ready-to-use predictor.

## Materials and Methods

### *Original* profile kernel

**Figure 1:**
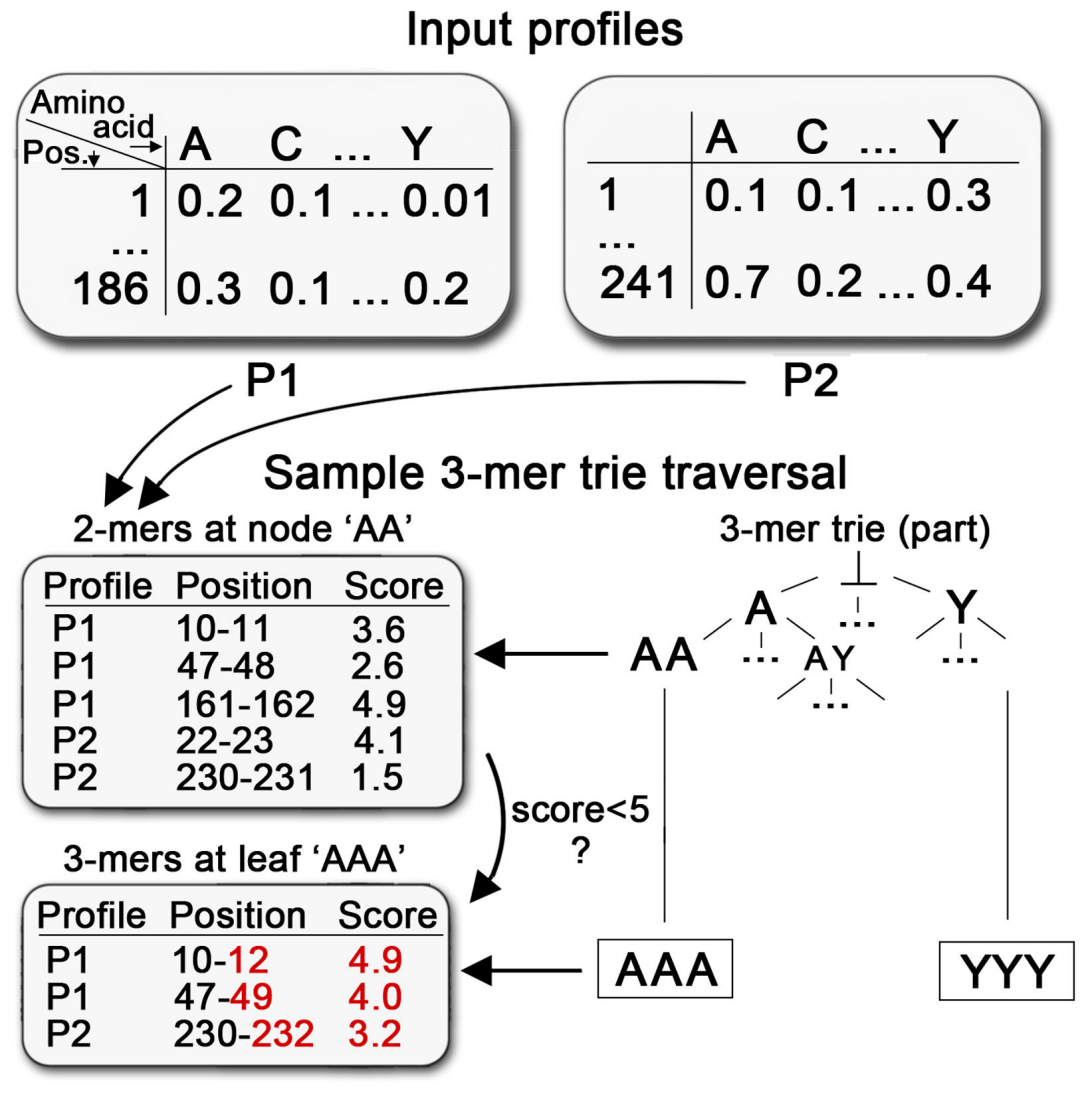
Sample k-mer tree traversal. Sketched is one part of a 3-mer trie traversal with two input profiles (P1 and P2). These profiles were generated with proteins that were 186 (P1) and 241 residues long (P2; tables on the top). During traversal, some conserved multi-mers remain at each node that fall below the substitution score threshold σ. The ‘Sample 3-mer trie traversal’ illustrates the transition from two-letter node ‘AA’ to node ‘AAA’ (‘AAA’ is also a leaf, because k=3). At node ‘AA’, five 2-mers have remained from previous transitions (root -> ‘A’ -> ‘AA’) that still fall below the substitution score threshold σ=5. In the transition to node ‘AAA’, each such 2-mer is extended to a 3-mer and each score re-calculated (k-mer extension and new scores in red). 3-mers with a score > 5 are discarded (2/5) and those that remain (3/5) are used in the kernel matrix update. Afterwards, the traversal continues until reaching the lexicographically last leaf (‘YYY’).

The algorithm to calculate the kernel matrix with the *original* profile kernel has been thoroughly introduced in [13]. It maps every profile to a 20^*k*-dimensional vector of integers. Each dimension represents one *k-mer* of *k* consecutive residues and a particular value gives the number of times this *k-mer* is conserved in a profile of related proteins. Conservation is calculated as the sum of the substitution scores for each residue in the *k-mer* profile and has to fall below a certain user defined threshold σ. Conserved *k-mers* are found very efficiently by traversing a trie based data structure (Figure 1). Each leaf corresponds to one of the 20^k dimensions and defines a set of conserved *k-mers*. With this set, the kernel matrix is updated so that each kernel matrix value is increased by the number of *k-mers* shared by the two corresponding profiles at that leaf.

In the following, we describe our own modifications and extensions to this approach. Technical details are given in Text S1. Our speed-up focuses on two different steps in the profile kernel algorithm: the trie traversal and the matrix update. Combined, these two always account for about 90% of the overall runtime, but their individual fraction depends on the respective kernel parameters and input. On average, we estimate that the two contribute equally to the runtime.

### Modification 1: Reducing kernel matrix updates to matrix multiplication

At each leaf node during the traversal of the *k-mer* trie, a set of conserved *k-mers* of the input profiles has remained (Figure 1). At this point, the original profile kernel updates the kernel matrix: if, e.g., *k-mer* 1 belongs to input profile 3 and *k-mer* 2 to input profile 8, then the value of the kernel matrix at row 3, column 8 has to be increased by 1. Repeating this for all *k-mer* pairs updates the entire kernel matrix for this particular leaf node and the traversal continues. This operation can be greatly simplified: first, we count how many conserved k-mers each profile has at a particular leaf node. Only the profiles with non-zero counts are added to a sparse matrix in which each row stands for a profile and each column for a particular leaf. (To save space, the matrix is stored as a “coordinate list”, i.e. as a list of triplets of the form [*x-coordinate, y-coordinate, value*].) For most leaves, we only add elements to this sparse matrix; only when the buffer is almost full, we update the actual kernel matrix. This can be done in arithmetically the same way as described above, but operationally by a very efficient self-multiplication of the buffered sparse matrix and an on-the-fly addition of the result to the kernel matrix (mathematical details in Section 1.1 of Text S1).

### Modification 2: SSE2 instructions and new data structure during tree traversal

Profiling the profile kernel executable with *perf* (part of the Linux kernel) revealed that during traversal of the k-mer trie, most of the time is spent on checking whether the substitution score of the *k-mers* is below the user-defined threshold. Implementing this double comparison with Streaming SIMD Extensions 2 (SSE2) instructions, two values can be compared in one CPU cycle, thus significantly improving overall runtime.

### Modification 3: Multi-process kernel matrix calculation

Too large kernel matrices can no longer be kept in main memory and may require several days for computation on a single CPU. Therefore, we have added the feature to split this task among several individual processes. Given *m* training profiles, we first assign each to one of *n* groups of size *p*=*m*/*n* (*n* is user defined). Then we compute the dot products of the profiles for one group to those of another group. This creates a *p* x *p* sub-matrix of the original kernel matrix. Repeating this for all O(*p* ^*2*^) possible group pairs calculates all sub-matrices which then have to be joined together to build the original kernel matrix. The creation of a single sub-matrix can be accelerated by only computing dot products between profiles from different groups and again by applying Modifications 1 and 2 (Sections 1 and 2 in Text S1 for mathematical details).

### Modification 4: Predicting new queries through normal vectors (application of model)

In contrast to the kernels described elsewhere [16], the original profile kernel introduced by the Leslie group allows the explicit calculation of the discriminative normal vector *w* of a SVM. The ‘SVM score’ of a new query profile*p*, i.e. its scaled distance to the hyper-plane, can then be calculated as a single dot product *s*=*w·Φ*(*p*), where *Φ*(*p*) is the feature vector of *p* and *Φ*(*p*)_*j*_ the number of conserved k-mers at leaf node *j*. In the original implementation, dot products to all support vectors were required (Section 2.1 in Text S1).

In order to extract normal vectors from trained SVMs, we can again use the k-mer trie. A single traversal can determine the normal vectors of many SVMs and create a ‘normal matrix’ in which each row represents one of 20^k *k-mers* and each column one normal vector (details in Section 2.1 of Text S1). This greatly accelerates the additional training time, as classification problems are hardly ever limited to two classes in computational biology.

In order to calculate the SVM score *s*=*w·Φ*(*p*) of a single query *p* and a single normal vector *w*, we multiply *w*_*j*_ with *Φ*(*p*)_*j*_ at each leaf node *j* and add the result to *s* (*s* is initialized to 0). By using the normal matrix (above), this can be modified so that the scores of all SVM normals are updated at each leaf node, resulting in a vector of SVM scores for query *p*. Traversing the trie with multiple queries at once consequently generates a matrix of SVM scores in which each row represents a target profile and each column a SVM.

With another extension similar to Modification 1, we can again store k-mer counts in a sparse matrix and use matrix multiplication to update the SVM scores matrix (Section 2.2 of Text S1). SSE2 instructions again accelerate the transition from one node to the next (Modification 2).

### Modification 5: Pipelining the training and prediction process

Using both, the normal and the SVM score matrices described above, renders training and applying a multi-class profile kernel based classifier a tedious task that requires many data management steps. We have therefore pipelined the entire model creation and application workflow in a Perl script. In “model creation” mode, it calculates the kernel matrix, uses it to learn an SVM multi-class classifier, extracts all weights for the Support Vectors from the resulting binary SVMs, converts these vectors into a matrix of normal vectors and stores all files and parameters that are required for predictions in a “model” folder. The user only has to provide the input profiles with class labels and to specify the kernel parameters, a Weka [21] multi-class model and the number of processes to use. The “model application” mode then uses this model to first calculate SVM scores with the normal matrix and the profile kernel and then forwards them to Weka which finally calculates the class probabilities of the queries.

### Modification 6: Predicting new targets with support vectors (baseline predictor)

In the original implementation of the profile kernel, there is no prediction mode. In order to classify a query, its profile has to be added to those of all support vectors and the kernel matrix has to be re-calculated. Comparing the impact of our modifications to this approach would be unfair, because a simple prediction mode can easily be added: first, the kernel matrix updates can be restricted to dot products between targets and support vectors only; secondly, at each node in the *k-mer* trie, we can stop going down further in the trie as soon as there are no more *k-mers* left that belong to the queries. Another difference to normal matrix based predictions (Modification 5) is the output of dot products to support vectors instead of SVM scores. This can be neglected, however, because the time needed by external multi-class classifiers to calculate SVM scores given dot products is minimal. In the following, we will refer to this slightly altered original implementation as the “baseline” implementation.

### Data sets

In order to measure the runtime improvement of our new implementation, we use four different data sets for kernel matrix computations and three for classifying new queries. They are described in detail in Section 5 of Text S1. In the following, we only give a short overview. All profiles are taken from a redundancy reduced Swiss-Prot database and readily available as part of the PredictProtein [22] cache.

The four training data sets correspond to 5920 profiles assigned to 18 classes (set “Euka (5920)”), 12,500 profiles assigned to 125 classes (set “SP60_25k”), 25,000 profiles assigned to 250 classes (set “SP60_25k”) and 100,000 profiles assigned to 1000 classes (set “SP60_100k”).

The runtimes for classifying new profiles were measured with models created from these four training data sets. As queries, we used three other data sets containing 1, 200 and 20,000 non-redundant protein profiles. They simulate typical classification tasks, ranging from the frequent single-user single-target case to the prediction of an entire genome.

## Results and Discussion

### Speed measurements under stringent conditions

We measured the impact of our modifications on the speed of both, the kernel matrix creation and the final application of the model, i.e. the prediction of new queries. The time needed to generate profiles was not included (Section 6 of Text S1 for a discussion). The baseline for kernel matrix computations was the original and publicly available profile kernel implementation from the Leslie lab (http://cbio.mskcc.org/leslielab/software/string-kernels); for predictions, we implemented the baseline ourselves (Methods: Modification 6). None of our modifications changed the original kernel arithmetically; the chance that floating point imprecisions will yield different classifications is very small, much less than 1:10^^^6. Also smaller changes of SVM scores are quite rare (1:10^^^4 for 0.01% change; Section 4 of Text S1). Therefore, all previously published values for accuracy remain valid.

Experiments were conducted on a 2 x 6-Core AMD Opteron Processor 2431 (2.4 Ghz) with 32GB DDR2 main memory using various data sets (Methods). Each kernel run was executed as the only active process on the entire computer, so that the conditions with respect to memory, disk and hyperthreading were similar for all experiments. Repeating the same measurements 20-30 times revealed a universal runtime standard error below 5%. The profile kernel has two free parameters: the length of the *k-mer* (k) and the substitution score threshold σ. Parameter combinations were taken from the original publication [13] and LocTree2 [20]. To our knowledge, only the latter optimized these parameters and found it preferable to use substantially higher substitution score thresholds than reported originally (“*k*=5, σ=9” and “*k*=6, σ=11”). Other papers using the profile kernel appeared to have copied the combinations reported in the original publication.

### Kernel matrix creation five times faster and parallelizable

**Figure 2:**
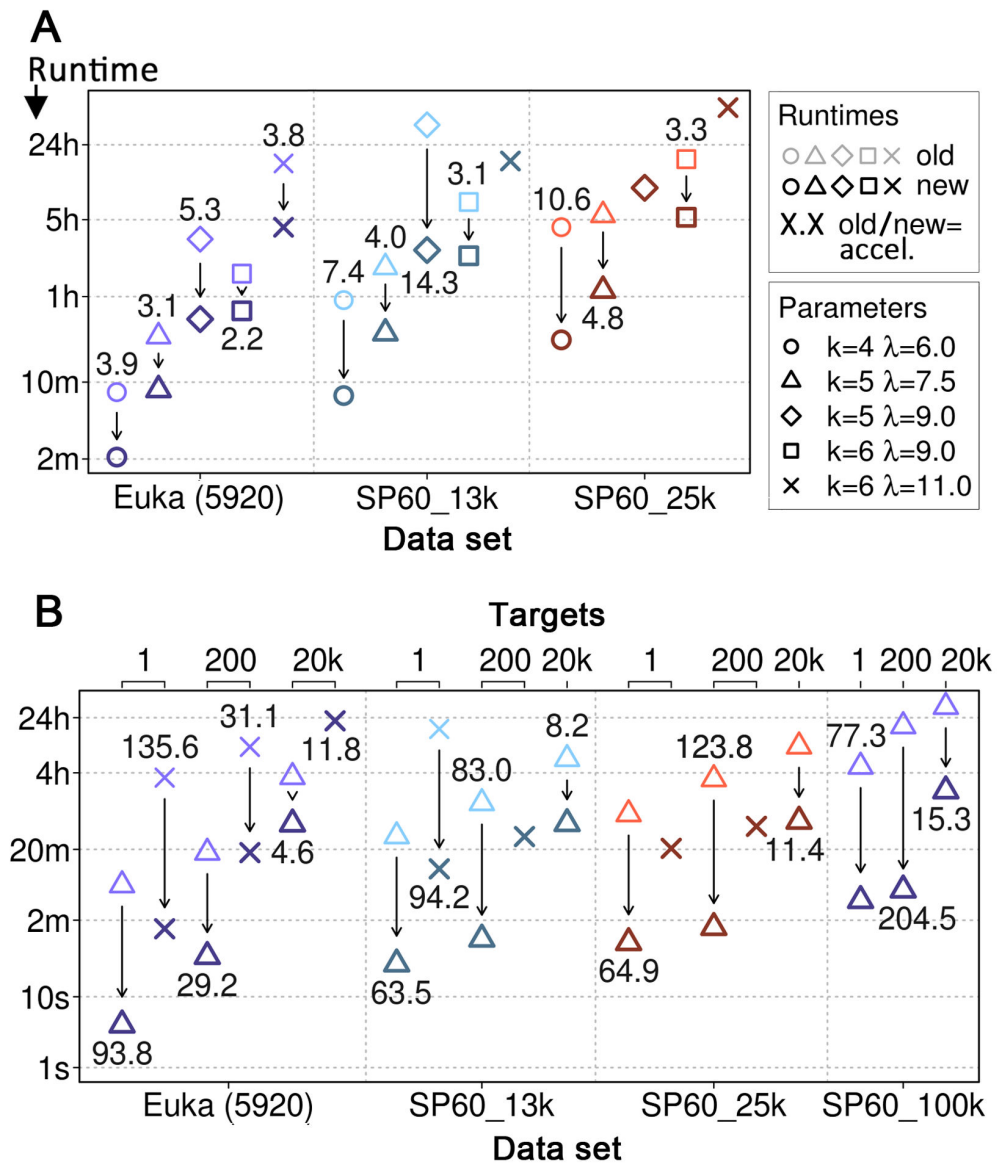
Speed measurements. Each arrow compares the runtime of the original implementation (upper symbol) to the new implementation (lower symbol). The symbol type indicates the parameter combination. The number above or below an arrow is the acceleration (original runtime divided by new runtime). All runtimes are wall-clock times of single processes. We did not perform an experiment if it was clear that it would take longer than 24 hours. (**A**) **Kernel matrix calculations**. In this subfigure we compare kernel matrix creation runtimes. Data sets correspond to subsets of a redundancy reduced Swiss-Prot database with 5920 (‘Euka (5920)’), 12,500 (‘SP60_13k’), 25,000 (‘SP60_25k’) and 100,000 (‘SP60_100k’) samples, respectively. The SP60_100k experiment (“k=5, σ=7.5”) for which we used 100 CPUs in parallel took 40 minutes and is not shown. (**B**) **Prediction of new targets**. This subfigure displays the runtimes for predicting three sets of targets (1, 200 and 20,000 profiles; axis on top) using models created with the training data sets (‘Euka (5920)’ to ‘SP60_100k’; axis on bottom).

Modifications 1 and 2 (Methods) yielded a constant acceleration, ranging from twice to up to 14 times faster with respect to the original implementation (Figure 2A). On average, the new implementation was about five times faster, with the speed-up increasing proportionally to the data set size. The kernel matrix computation for the SP60_100k data set (Methods) no longer fit into the main memory of our machine (approx. 56GB). Hence, we used our new splitting technique (Methods; Modification 3) to distribute its calculation amongst 100 individual processes that were run simultaneously on a computer cluster (the CPU conditions described in the paragraph above no longer applied for this proof-of-concept run). This took about 40 minutes.

The speed of the kernel critically depends on its two parameters (Figure 2). The large difference between, e.g. “*k*=6, σ=9” and “*k*=6, σ=11”, is due to a loss of sparseness and an accumulation of conserved *k-mer*s during the trie traversal. However, in our hands, this actually improved performance for the development of LocTree2 [20], suggesting a relative enhancement of the conserved *k-mer* signal despite a probable increase of background noise. Indeed, we found the feature vectors resulting from “*k*=6, σ=11” to be sparse but less so than those resulting from training with “*k*=6, σ=9”.

### Predictions accelerated by orders of magnitudes

Besides a general code optimization, our modifications include the feature to calculate the SVM scores for many queries and SVMs in one profile kernel run (model application mode; Methods: Modifications 4 and 5). We compare this variant to the original implementation extended by a support vector based application mode (Methods: Modification 6). The normal vector based variant that we introduced here, is at least five times faster than the support vector based alternative (Figure 2B, Euka data set, 20,000 targets, “*k*=5, σ=7.5”), with a maximum acceleration of 205-fold (Figure 2B, SP60_100k, 200 target, “*k*=5, σ=7.5”). On average (arithmetic mean over all experiments), our new implementation turned out to be about 66 times faster than the original implementation. Again: for larger data sets, the speed-up would increase.

As long as the models are queried only with a few targets (up to about 200), the most limiting factor is the size of the normal vector matrix. For *k*=5, even the matrix with 1000 SVMs still remains below 10GB (8.2GB), but it grows to 39GB for *k*=6 and 250 classes and consequently takes about 20 minutes to be read from disk.

### Comparison to SVM-Fold and SW-PSSM

Generating the same output as the original version, our new profile kernel implementation can directly be used in existing profile kernel based classifiers such as SVM-Fold [23]. This web-server predicts SCOP classes from protein sequence. Multiple binary SVMs are trained and embedded in a multi-class scheme, called ‘adaptive codes’, which exploits the hierarchical structure of SCOP. Extending or replacing the Weka-based multi-class models with the adaptive codes approach, our new workflow script (Methods; Modification 5) could generate SVM-Fold automatically. For predictions, SVM-Fold uses the baseline implementation (Methods; Modification 6) with an additional caching of k-mers in the higher levels of the k-mer trie. Prediction speed could be greatly increased by using pre-computed normal matrices (Methods; Modification 4).

A popular competitor of the original profile kernel in terms of classification accuracy is SW-PSSM [16] (Smith-Waterman Position Specific Scoring Matrix). We have compared our implementation of the original profile kernel to this method and found our program to be multiple orders of magnitudes faster (Section 7 of Text S1 for details).

### Future accelerations

Our new profile kernel implementation could be accelerated even more. Future releases might include the following improvements.

Optimizing a classifier requires evaluating alternative kernel matrices that only differ by the parameters with which they were created (*k* and σ). The matrices can all be calculated in a single trie traversal. For example, with alternative parameters “*k*=4, σ=6”, and “*k*=6, σ=9”, the cumulative substitution score of a k-mer only has to be compared to nine at any node with a depth <4 and >4. Only at a node of depth 4 (a leaf node for k=4), it additionally has to be checked against 6 in order to correctly update the kernel matrix for parameters “*k*=4, σ=6”. Afterwards, the traversal continues with threshold 9 until reaching the maximum depth (6). This principle can be extended to an arbitrary amount of parameter combinations and should greatly reduce the number of double comparisons during trie traversal. On the implementation side, it requires an in-memory kernel matrix and a sparse matrix buffer for each parameter combination.

For the prediction of new queries (application mode), the most significant bottleneck is reading and uncompressing the normal matrix (before). Novel types of disks (e.g. solid state drives) and decompression algorithms (e.g. Google’s lz4) might yield another 5-fold acceleration on top of what we have presented here. Given the appropriate hardware, the matrix might also be kept in memory, thus practically eliminating the bottleneck.

## Conclusion

The original profile kernel proposed by the Leslie group is highly accurate and can be applied to many classification problems. Our new implementation produces the identical results with considerably fewer computer resources (in terms of runtime and memory).

It is available as a Debian package under a free academic license and without dependencies on commercial products. All Debian-based Linux systems (Ubuntu, Xandros, Mint,…) may install it via their respective package managers. For all other systems, the source package features a make-based compilation and installation. Detailed instructions and download links can be found at https://rostlab.org/owiki/index.php/Fast_Profile_Kernel. Bugs may be reported via https://rostlab.org/bugzilla3/enter_bug.cgi?product=fastprofkernel. For documentation, we have written man pages that are shipped with the package, as is a small sample classification problem.

The package installs two new executables: “profkernel-core” and “profkernel-workflow”. The first is our new, backward-compatible implementation of the original profile kernel. All parameters and output formats of the original release by the Leslie group have been preserved. The second is a Perl script that uses this binary and its new features as part of a model creation and application workflow. It can both automatically create new models and apply them to new queries.

## Supporting Information

Text S1 Mathematical details and additional information. This supporting text provides mathematical details about the profile kernel accelerations and the matrix multiplication algorithms. Additional studies investigate the possible precision loss of the new implementation due to floating point operations and compare runtimes to SW-PSSM. Finally, we describe the data set in more detail and discuss whether profile generation runtimes should be considered when measuring kernel speed.
(PDF)
