# Supplementary material for: Accelerating the Original Profile Kernel
Source: PLoS One. 2013 Jun 18;8(6):e68459. doi: 10.1371/journal.pone.0068459 (PMC3688983; doi:10.1371/journal.pone.0068459)
Supplement: Text S1 — This supporting text provides mathematical details about the profile kernel accelerations and the matrix multiplication algorithms. Additional studies investigate the possible precision loss of the new implementation due to floating point operations and compare runtimes to SW-PSSM. Finally, we describe the data set in more detail and discuss whether profile generation runtimes should be considered when measuring kernel speed. (PDF) [file pone.0068459.s001.pdf]

Accelerating the original profile kernel

Supplementary Online Materials

Tobias Hamp, Tatyana Goldberg and Burkhard Rost

Contents

S1 Acceleration of the creation of the kernel matrix **2**
S1.1 Reducing kernel matrix updates to matrix multiplication 2
S1.2 Calculating a kernel submatrix 3

S2 Accelerating predictions **4**
S2.1 Calculating the matrix of normal vectors 4
S2.2 Applying the matrix of normal vectors 5

S3 Matrix multiplications **6**

S4 Equivalence of old and new implementation **7**

S5 Data sets **9**

S6 Discussion: profile generation and runtime measurements **10**

S7 Comparison to SW-PSSM **11**

S1 Acceleration of the creation of the kernel matrix

S1.1 Reducing kernel matrix updates to matrix multiplication

Let $\mathbb{P}$ be the space of all possible protein profiles and $p_1, ..., p_m \in \mathbb{P}$ be the input protein profiles. Let $length : \mathbb{P} \rightarrow \mathbb{N}$ be a function that returns the length of each profile (i.e. the length of the sequence that a profile p_i was generated with). Let further $Kmers = \{(l, r) | 1 \leq l \leq m \wedge 1 \leq r \leq length(p_l) - k + 1\}$ be the set of all k-mer starting positions with k being the user-defined k-mer length and let $A = [a_{i,j}]_{m \times m}$ be the kernel matrix.

At a leaf node L, there remains a set $Kmers_L \subset Kmers$ of k-mers whose cumulative substitution score is lower than a pre-defined threshold [1]. In the original profile kernel implementation, the subsequent kernel matrix update is performed in the following way:

for all $(l, r) \in Kmers_L$ **do**
    for all $(l', r') \in Kmers_L$ **do**
        $A_{l,l'} \leftarrow A_{l,l'} + 1$
    end for
end for

By definition, a kernel matrix value $A_{l,l'}$ is the result of a dot product $\phi(p_l)\phi(p_{l'})$ and one of the 20^k dimensions of any $\phi(p_i)$ is processed at every leaf node. However, the implementation above never calculates a dimension of a $\phi(p_i)$ explicitly, but directly updates the kernel matrix. Hence, it could even be argued to be a 'kernel trick', which is commonly regarded as an acceleration of a kernel. In the following, however, we show how its replacement with the explicit feature mapping and the subsequent reduction to matrix multiplication leads to a more efficient procedure.

First, note that r and r' are never used and can be ignored. Secondly, after one entire inner **for all** loop, the value $A_{l,l'}$ has been increased by the number of k-mers that have remained from profile $p_{l'}$. This number can be calculated as $s^L(l') = \sum_{(l,r) \in Kmers_L} I(l = l')$, where $I(x)$ is 1 if x is true and 0 otherwise, and allows re-writing the matrix update in the following way:

$\mathbf{v^L} \leftarrow \langle s^L(1), ..., s^L(m) \rangle$
for all $(l, r) \in Kmers_L$ **do**
    for $j \leftarrow 1; j \leq m; j \leftarrow j + 1$ **do**
        $A_{l,j} \leftarrow A_{l,j} + v_j^L$
    end for
end for

In this new version, instead of iterating over $Kmers_L$ in the inner loop, we only iterate over the number of profiles. Since s^L only depends on $Kmers_L$ and not on A, it can be pre-computed before the matrix update and the time complexity of the latter is reduced from $O(|Kmers_L|^2)$ to $O(m \cdot |Kmers_L|)$. Now, note again that v_j^L is added to $A_{l,j}$ exactly as often as there are kmers

from profile l in $Kmers_L$. This means we can also re-write the outer for loop:

$\mathbf{v^L} \leftarrow \langle s^L(1), ..., s^L(m) \rangle$
for $i \leftarrow 1; i < m; a \leftarrow i+1$ **do**
  for $j \leftarrow 1; j < m; b \leftarrow j+1$ **do**
    $A_{i,j} \leftarrow A_{i,j} + v_i^L \cdot v_j^L$
  end for
end for

Obviously, the time complexity for this operation is now $O(m^2)$. Next, assume that the matrix update is not executed at every leaf node, but instead only every x leaf nodes ($1 \leq x \leq 20^k$). At leaves without an update, the vector $\mathbf{v^L}$ is added to a matrix $\tilde{V}$ as a new row. When reaching an update leaf, $\tilde{V}$ has the form $\tilde{V} = [\tilde{v}_{i,j}]_{x \times m}$ and the bulk matrix update can be performed in the following way:

for $i \leftarrow 1; i < m; i \leftarrow i+1$ **do**
  for $j \leftarrow 1; j < m; j \leftarrow j+1$ **do**
    $A_{i,j} \leftarrow A_{i,j} + \tilde{V}_{1,i} \cdot \tilde{V}_{1,j} + ... + \tilde{V}_{x,i} \cdot \tilde{V}_{x,j}$
  end for
end for

Simplifying even further, we obtain: $\tilde{V}_{1,i} \cdot \tilde{V}_{1,j} + ... + \tilde{V}_{x,i} \cdot \tilde{V}_{x,j} = \tilde{V}^T_{i,1} \cdot \tilde{V}_{1,j} + ... + \tilde{V}^T_{i,x} \cdot \tilde{V}_{x,j} = (\tilde{V}^T\tilde{V})_{i,j}$ and the entire matrix update reduces to matrix multiplication:

$A = A + \tilde{V}^T\tilde{V}$

This operation can be implemented very efficiently with a slight modification of the IKJ algorithm introduced in [2] so that matrices are stored in a sparse format and cache efficiency is preserved (Section S3 for more details). As each value $(\tilde{V}^T\tilde{V})_{i,j}$ is calculated separately by the algorithm, there is also no need for a temporary matrix to store $\tilde{V}^T\tilde{V}$. $(\tilde{V}^T\tilde{V})_{i,j}$ can be added directly to the kernel matrix. In our actual implementation, $\tilde{V}$ is a triplet-based sparse matrix with a maximum size of 300MB. In order to make sure that every row addition still fits into memory, the current matrix size is checked at every leaf node and an update is performed as soon as more than 67% of the buffer are used or the last leaf is reached. After the matrix update, the buffer is emptied and the traversal continues until all leaves are reached.

Interpreting the above in terms of the explicit feature mapping ϕ, we calculate one of the 20^k dimensions of every feature vector $\phi(p_i)$ at each leaf node L and store it in $\tilde{V}$. Before a kernel matrix update, $\tilde{V}$ contains x dimensions of every $\phi(p_i)$ and the actual matrix update calculates all of their pairwise dot products.

S1.2 Calculating a kernel submatrix

In certain situations, only a consecutive submatrix $A'[a_{i,j}]_{n \times n'}$ of A has to be calculated, i.e. $A'_{i,j} = A_{n_{start}+i-1, n'_{start}+j-1}$ where n_{start} denotes first row in A and n'_{start} the first column.

A certain value $A_{i,j}$ only depends on the columns i and j of matrix $\tilde{V}$. Hence, in order to reduce $\tilde{V}$ to the required columns for A', we project it to two submatrices $\tilde{V}' = [\tilde{v}_{i,j}]_{x \times n}$ and $\tilde{V}'' = [\tilde{v}_{i,j}]_{x \times n'}$ with $\tilde{V}'_{i,j} = \tilde{V}_{i,n_{start}+j-1}$ and $\tilde{V}''_{i,j} = \tilde{V}_{i,n'_{start}+j-1}$. Submatrix A' can then be calculated as:

for $i \leftarrow 1; i < n; a \leftarrow i + 1$ **do**
  for $j \leftarrow 1; j < n'; j \leftarrow j + 1$ **do**
    $A'_{i,j} \leftarrow A'_{i,j} + \tilde{V}'_{1,i} \cdot \tilde{V}''_{1,j} + ... + \tilde{V}'_{x,i} \cdot \tilde{V}''_{x,j}$
  end for
end for

This can be simplified to:

$A' = A' + \tilde{V}'^T \tilde{V}''$

In practice, $\tilde{V}'$ and $\tilde{V}''$ can be calculated directly by accepting two sets of input profiles $p \subset \mathbb{P}$ and $p' \subset \mathbb{P}$ that correspond to the rows and columns of A', respectively. When traversing the trie, conserved k-mers of p are added to $\tilde{V}'$ and conserved k-mers of p' to $\tilde{V}''$, as described in Section S1.1 for $\tilde{V}$.

S2 Accelerating predictions

S2.1 Calculating the matrix of normal vectors

With one profile kernel matrix, several binary Support Vector Machines (SVMs) can be trained. The exact number of SVMs depends on the number of classes and the multi-class scheme. For a traditional 1-vs-All classifier, e.g., we train as many SVMs as there are classes.

Classifying a new target profile p_{m+1} requires calculating its scaled distance to the SVM hyperplanes. One distance is computed with the well-known formula $s_{m+1} = w \cdot \phi(p_{m+1}) = \sum_{i=1...m} \alpha_i \phi(p_i) \phi(p_{m+1})$ where m is the number of support vectors, α_i the weight, p_i the original profile and $\phi(p_i)$ the feature vector of support vector i. This means s_{m+1} can either be calculated via support vectors (Methods; Modification 6) or via a pre-computed normal vector w. In the following, we describe how to use the k-mer trie traversal to efficiently calculate several of these normal vectors in one run with support vector profiles and associated α's as input. In the next Section, we apply this matrix of normal vectors to predict new queries.

Let $\mathbb{P}$ be the set of all possible protein profiles and $length : \mathbb{P} \rightarrow \mathbb{N}$ be a function that returns the length of each profile (i.e. the length of the sequence that a profile p was generated with). Let $(p_1, ..., p_m) \in \mathbb{P}^m$ be the original training profiles and $T = [t_{i,j}]_{m \times n}$ be the matrix of support vector weights extracted from n pre-computed SVMs, where each SVM corresponds to a column and each row to a training profile. One value $T_{i,j}$ indicates the weight of profile p_i in SVM j (the weights of non-support vectors are set to 0). Let $Q = \{i | 1 \leq i \leq m \wedge \exists j : T_{i,j} \neq 0\}$ be the indices of those training profiles that appear as a support vector at least once. Let $q = (q_i)_{q_i \in Q}$ be Q as an ordered tuple and let $m' = |Q|$ be the size of Q. Let further $Kmers = \{(l, r) | l \in Q \wedge$

$1 \leq r \leq length(p_l) - k + 1\}$ be the set of all k-mer starting positions of all support vector profiles. Our goal is to compute a matrix $W = [w_{i,j}]_{20^k,n}$ where each $W_{i,j}$ indicates the value of the i-th dimension in the normal vector of SVM j and is defined as $W_{i,j} = \sum_{z=1...m'} T_{q_z,j}\phi(p_{q_z})$ (follows directly from $w = \sum_{i=1...m} \alpha_i \phi(p_i)$). Once W is calculated, each column j corresponds to the normal vector of SVM j.

We start the trie traversal with all support vector profiles p_{q_i} and matrix T as input. At a leaf node L, there remains a set $Kmers_L \subset Kmers$ of k-mers whose cumulative substitution score is lower than a pre-defined threshold. This set $Kmers_L$ is converted to a vector v^L of size m so that $v_i^L = \phi(p_{q_i})_L = \sum_{(l,r)\in Kmers_L} I(l = q_i)$ (analogously to Section S1.1). One row W_L is then defined as $W_{L,j} = \sum_{z=1...m'} T_{q_z,j} v_z^L$.

As W can become very large and sparse, we only store non-zero entries of each row and write it directly to a file. As soon as it grows over a certain size, it is compressed with zlib [3] (DEFLATE compression [4]) and the output is directed to a new file.

S2.2 Applying the matrix of normal vectors

In order to classify a new profile p_{m+1} (m is the number of training samples) with a single SVM, we need to calculate its scaled distance to the hyperplane. This distance, which will be referred to as the 'SVM score', is given by the formula $s_{m+1} = w \cdot \phi(p_{m+1})$, where w is the normal vector of the SVM and $\phi(p_{m+1})$ the feature vector of profile p_{m+1}. In practice, often many samples have to be classified by many SVMs at the same time, e.g. to predict all proteins of a genome in a multi-class setting. In the following, we show how to achieve this very efficiently in a single k-mer trie traversal and using the matrix of pre-computed normal vectors (previous Section).

Let $\mathbb{P}$ be the set of all possible protein profiles. Let $length : \mathbb{P} \rightarrow \mathbb{N}$ be a function that returns the length of each profile (i.e. the length of the sequence that profile p_i was generated with) and $(p_1, ..., p_m) \in \mathbb{P}^m$ be the target profiles. During the trie traversal, we use a matrix $\tilde{V}$ as a buffer which stores dimensions x_{start} to x_{end} of all $\phi(p_i)$'s (x_{start} and x_{end} are initialized to 0 and x, respectively; x is user-defined). Let $W = [w_{i,j}]_{20^k \times n}$ be the matrix of normal vectors, where k is the user-defined k-mer length and n the number of SVMs. Each column of W corresponds to a normal vector and each row to a k-mer. As W is usually too large to be kept entirely in memory, we use a temporary matrix $\tilde{W} = [\tilde{w}_{i,j}]_{x \times n}$ to store only the rows of W currently needed. Our goal is to calculate matrix $\tilde{D} = [d_{i,j}]_{m,n}$ where each $D_{i,j}$ indicates the SVM score of profile p_i after classification by SVM j, i.e. $D_{i,j} = w^j \cdot \phi(p_i)$, where w^j is the normal vector of the j-th SVM.

The trie traversal begins with all support vector profiles p_i. At a leaf node L, there remains a set $Kmers_L \subset Kmers$ of k-mers whose cumulative substitution score is lower than a predefined threshold. This set $Kmers_L$ is converted to a vector v^L of size m so that $v_i^L = \phi(p_i)_L = \sum_{(l,r)\in Kmers_L} I(l = i)$ (identical to

previous Sections). Each vector v^L is added to matrix $\tilde{V}$ as a new row. When rows x_{start} to x_{end} have been added, we load rows of matrix W with the same indices into $\tilde{W}$ and perform the following procedure:

for $i \leftarrow 1; i < m; i \leftarrow i + 1$ **do**
  for $j \leftarrow 1; i < n; j \leftarrow j + 1$ **do**
    $D_{i,j} \leftarrow D_{i,j} + \tilde{V}_{1,i} \cdot \tilde{W}_{1,j} + ... + \tilde{V}_{x,i} \cdot \tilde{W}_{x,j}$
  end for
end for

With a proof analogous to that of Section S1.1, this procedure reduces to

$D \leftarrow D + \tilde{V}^T \tilde{W}$

The matrix multiplication is carried out with the Eigen package ([5]; Section S3 for more details). After the matrix update, the value of x_{end} is assigned to x_{start} and x_{end} is adjusted to fit the next update of D. $\tilde{V}$ and $\tilde{W}$ are emptied.

The above calculates the dot product between profile i and normal vector j, reduced to the summands x_{start} to x_{end} and adds the result to $D_{i,j}$. As the range $[x_{start}, x_{end}]$ has contained every k-mer index exactly once after the trie traversal, we obtain $D_{i,j} = w^j \cdot \phi(p_i)$ in the end.

S3 Matrix multiplications

In the previous Sections, we either had to multiply a sparse matrix with a dense matrix or a sparse matrix with a sparse matrix. In both cases, the result had to be added to another third matrix.

For sparse-dense multiplications, we used the operations provided by the Eigen package [5]. For sparse-sparse multiplications, however, all Eigen operations resulted in at least one temporary matrix and a significant additional memory overhead. As the result of sparse-sparse multiplications is as big as the kernel matrix, we found this to be unacceptable and implemented the operation ourselves. As a template, we used the IKJ algorithm detailed in [2]. This algorithm described the cache efficient multiplication of a sparse matrix with a dense matrix, but we extended it to the sparse-sparse case. It turned out to not only save memory but also CPU time in comparison to Eigen.

So far, we stored sparse matrices as coordinate lists. Before a multiplication, we convert them to the following format (runtime and space complexity of the conversion is trivially linear in the number of coordinates).

- $M_{sparse} \rightarrow$ *data*: an array of all non-zero data values of M_{sparse} in row-major format, i.e. the series of non-zero values obtained by reading the first row of M_{sparse} from left to right and then repeating the same for the second, third, ... row (exactly as in [2]).

- $M_{sparse} \rightarrow$ *index_col*: an array of column indices, with one index for each value in $M_{sparse} \rightarrow$ *data*. For example, if the first two non-zero elements of M_{sparse} are in row one, columns three and five, then the first two values of $M_{sparse} \rightarrow$ *index_col* are 2 and 4 (exactly as in [2]).

- $M_{sparse} \rightarrow index\_row$: an array of offsets where each offset points to the first element of a row in $M_{sparse} \rightarrow data$, in row-major format. For example, if the first two rows of M contain three and seven elements, then the first three values of $M_{sparse} \rightarrow index\_row$ are 0, 3 and 10 (3+7; new).

- $M_{sparse} \rightarrow length$: an array containing the counts of non-zero data elements of each row, in row-major format. For example, if the first two rows of M_{sparse} contain three and seven elements, the first two values of $M_{sparse} \rightarrow length$ are 3 and 7 (exactly as in [2]).

Note that $M_{sparse} \rightarrow data$ and $M_{sparse} \rightarrow index\_col$ have the same length (the number of non-zero elements in M_{sparse}), as do $M_{sparse} \rightarrow length$ and $M_{sparse} \rightarrow index\_row$ (the number of rows in M_{sparse}).

$M_{sparse} \rightarrow index\_col$, $M_{sparse} \rightarrow index\_row$ and $M_{sparse} \rightarrow length$ only need (short) integer data types, however.

We can now perform the sparse-sparse matrix multiplication and the on-the-fly addition of the result to a third dense matrix in the following way.

procedure MULTIPLYANDADD(A, B, R)

$A \leftarrow$ the first sparse matrix of size $m \times n$
$B \leftarrow$ the second sparse matrix of size $n \times m$
$R \leftarrow$ the dense matrix

$index_A = 0$
for $i = 0; i < m; i = i + 1$ **do**
  $len_A^{row} = A \rightarrow length[i]$
  for $k = 0; k < len_A^{row}; k = k + 1$ **do**
    $col_A = A \rightarrow index\_col[index_A + k]$
    $data_A = A \rightarrow data[index_A + k]$
    $len_B^{row} = B \rightarrow length[col_A]$
    $index_B = B \rightarrow index\_row[col_A]$
    for $j = 0; j < len_B^{row}; j = j + 1$ **do**
      $value_{old} = C[i][B \rightarrow index\_col[index_B + j]]$
      $value_{toAdd} = data_A \cdot B \rightarrow data[index_B + j]$
      $C[i][B \rightarrow index\_col[index_B + j]] = value_{old} + value_{toAdd}$
    end for
  end for
  $index_A = index_A + len_A^{row}$
end for
end procedure

S4 Equivalence of old and new implementation

Mathematically, both the old and the new implementation produce the same output for the same input. In practice, however, differences could arise due to

the imprecision of floating point numbers.

It has to be stressed that even the old implementation will not always produce the exact same results. The result of a double multiplication, for example, depends on the order of CPU instructions produced by the compiler. Even given the same compiler, different architectures and optimization levels will change this order. Another source for differences are downstream programs. The kernel matrix will be used in other algorithms, for example a support vector machine. Even different implementations of the same algorithm (e.g. Sequential Minimal Optimization [6]) will most likely generate different models.

Our changes to the generation of the kernel matrix have not added any additional source for differences. The comparison of cumulative substitution scores to the user-defined threshold is still carried out with 64 bit double precision types. The actual kernel matrix values are stored as integers and hence not subject to precision loss.

The question that remains is whether the additional (preprocessing) steps for predicting queries with our pipeline (Modification 5) has resulted in a loss of precision compared to the old baseline method (Modification 6). To answer this, we have predicted 2,000 random targets from our 20,000 targets test set with models created from the 'Euka (5920)' dataset (multi-class model: 'Nested Dichtomoy for Eukaryota' [7]; 17 SVMs; $k = 6$; $\sigma = 11$) in two different ways. One time, we used the original baseline implementation of the profile kernel to generate dot products which were then used as input for a Weka model. The latter processed these dot products in 17 precomputed SVMs to output 17 SVM scores for each query, i.e. scaled distances to the hyperplanes. The other time, we calculated these SVM scores with a normal matrix generated by our pipeline (Modification 5; pipeline used the same Weka model) and our new profile kernel implementation.

This resulted in $17 * 2,000 = 34,000$ value pairs that were mathematically identical, but could differ due to floating point imprecision. Next, we determined for each value pair their difference, which we call the 'distance fluctuation'. It corresponds to the difference in the distances to the SVM hyperplane. Then we divided the distance fluctuation by the distance of the supposedly correct distance, i.e. the distance calculated with the original implementation. The result, called 'relative distance fluctuation', can be interpreted as the degree with which the fluctuation influenced the actual decision made by the SVM. A value greater than 1.0 ($> 100\%$ change) means that the fluctuation changed the class of a sample point for this particular SVM (i.e. the side of the hyperplane the sample was on), a value below 0.01 ($< 1\%$ change) indicates that the fluctuation had virtually no impact. Fig. S1 shows the distribution of relative distance fluctuations.

None of the 34,000 relative distance fluctuations was greater than 0.001 and 4 were between 0.001 and 0.0001, which correspond to a frequency of 4/34,000≈0.0001. This means about 1 in 10,000 distances to the hyperplane will be at most 0.1% different from the actual value. Such an effect will not have a measurable impact on a classifier. For example, the 2,000 class probabilities calculated by the Weka multi-class model from the old and the new SVM

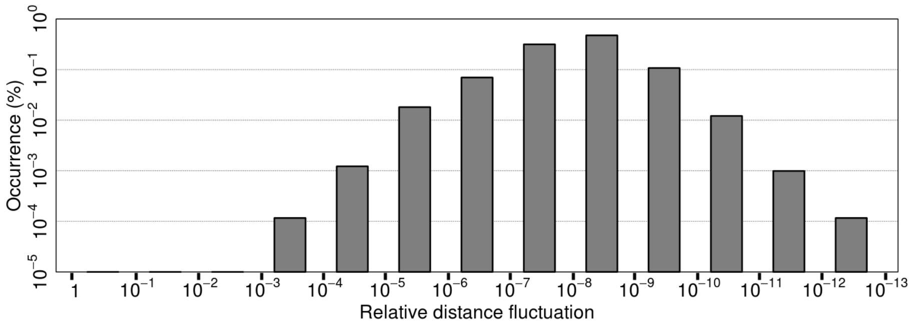

Figure S1: Distribution of relative distance fluctuations. This figure shows how much the new kernel implementation changed SVM scores compared to the old implementation. A 'relative distance fluctuation' is the difference between the old and the new score, divided by the old score. The plot is a histogram of 34,000 such fluctuations. Please see the text for how the SVM scores were calculated.

scores (one value for the 'winning' class in percentage, rounded to three decimal digits) were absolutely identical. (In case of multiple classes, even a dramatic change of a SVM score will most likely not result in a different classification due to the influence of other SVMs on the final class.)

To extrapolate probabilities of more severe changes, note that the occurrences in the outer bins of Fig. S1 decrease with about a factor of 10. A relative distance fluctuation between 0.1% and 1% will probably occur in about 1 of every 100,000 SVM decisions, and an actual hyperplane side change ($\geq$100%) in much less than 1 of every 1,000,000 SVM decisions.

S5 Data sets

Modifications 1 to 3 (main text) should significantly accelerate the computation of kernel matrices compared to the original profile kernel implementation. Similarly, Modification 5 is expected to speed up the classification of new query proteins, even if the original implementation is extended by a simple prediction mode (Modification 6). In order to obtain exact measurements, we created four different data sets for training and three for testing.

The first training set ("Euka (5920)") comes from the recent LocTree2 method [7] which predicts protein localization. It consists of 5920 eukaryotic proteins from the UniProt/Swiss-Prot [8] database, assigned to one of 18 sub-cellular localization classes based on experimental evidence. For the other three, we first redundancy reduced all Swiss-Prot sequences to 60% pairwise sequence identity using cd-hit [9] and then sub-sampled 12,500 ("SP60_13k"), 25,000 ("SP60_25k") and 100,000 ("SP60_100k") proteins. While irrelevant for kernel matrix computations, classes of training samples strongly affect predic-

tion speed as they determine the number of SVMs and Support Vectors. We estimated these parameters for the three larger data sets using the data of the final online version of LocTree2 and our own experience. For the LocTree2 data, 18 classes in a 1-vs-all schema (nuclear-vs-all, cytoplasm-vs-all, etc.) sufficed to render 84% of all training profiles Support Vectors. This implied that in order to classify one protein through the baseline method (Modification 6), dot products to 4954 other profiles have to be calculated. Given the 84% value, we assumed all profiles of the larger data sets to be support vectors in order to create realistic, large-scale classification problems. The speed of the normal vector based classification (Modification 4) depends on the number of SVMs. We assumed 125, 250 and 1000 SVMs for the three large-scale profile sets (SP60_13k, SP60_25k, and SP60_100k respectively), corresponding to about 100 proteins per class with a 1-vs-all or Nested Dichotomy [10] based multi-class schema. Note that this underestimates the relative performance of the normal vector based classification: considerably fewer classes should suffice to render almost all training samples Support Vectors and the class sizes in this example were smaller than those for LocTree2. Actual normal vector values were randomly sampled from the LocTree2 vectors, but have no impact on the speed of the application/prediction. For the testing sets, we again used the redundancy reduced Swiss-Prot database and sub-sampled 1, 200 and 20000 proteins (the one protein was a transpeptidase with 246 amino acids). We did not need class assignments for the testing sets, as we only measured prediction speed, not accuracy. The three sets simulate typical classification tasks, ranging from the frequent single-user single-target case to the prediction of an entire genome.

All profiles of all seven data sets were readily available as part of the PredictProtein [11] cache and created by three iterations of PSI-BLAST [12] against UniProt [13] (e-value 10^{-3}).

S6 Discussion: profile generation and runtime measurements

Profile kernels require the generation of a profile taken out of a multiple alignment of related proteins. This in turn has to be generated by alignment methods such as PSI-BLAST [12], HMMer [14], or HHblits [15]. Should the generation of these profiles be included in measuring the runtime for the profile kernels? We argue against this, primarily because such an approach still does not capture all tasks required for the creation and application of a model. For example, the knowledge of protein sequences does not come for free: Most proteins in UniProt/TrEMBLE [13] are predictions for the gene products, i.e. their sequences are inferred from intron-exon structures generated by a possibly very slow computational method. In UniProt/Swiss-Prot [8], protein existence is most often inferred by homology, implying that, e.g., BLAST [12] has already been run, exactly as needed to generate a profile. Hence, drawing a line between sequences and profiles appears unreasonable in context of measuring CPU us-

age. Furthermore, even if the time for profile generation had been included in the runtimes, simply adding it to the times we reported for the kernel might not estimate the costs completely correctly. Firstly, many profiles might be available directly from databases such as HHblits [15], or from our own Predict-Protein [11] cache that is used to feed LocTree2 [7], i.e. the first method using the new implementation. Secondly, during method training, multiple matrices are needed to find the best parameter combinations. For example, for LocTree2 [7], the optimal values for k and σ differed between the kingdoms and all values that we found to be optimal for this task differed from those reported in the original publication presenting generic examples. Thirdly, for predictions, a service-oriented architecture may be preferred, in which the user only provides the input and the actual computation is done on an external server. As running a search for homologs can be considered a standard procedure in most genetic experiments, requiring a profile from the user will not be a major hurdle.

S7 Comparison to SW-PSSM

SW-PSSM (Smith-Waterman Position Specific Scoring Matrix) [16] calculates a profile-profile alignment and converts its score into a valid kernel value. "Valid" means that it makes the score comply with Mercer's conditions [17], i.e. it turns finding the maximum margin hyperplane into convex optimization problem, implying that it does not have a local minimum. As the feature space is unknown, each value in the kernel matrix has to be calculated separately and only predictions based on the Support Vectors are possible. Using the publicly available implementation of SW-PSSM with the optimal parameter choices taken from elsewhere [16] and our own SP60 data set (Section S5), we found that the average time to compute the score of two profiles is 21 ms. Hence, creating a kernel matrix takes about 4.4 CPU days with 6000 samples, 19 CPU days with 12,500 samples and 3.3 CPU years with 100,000 samples Classifying a single target with the same number of Support Vectors takes about 2, 4, and 35 minutes, respectively. Comparing this to the runtime for our new implementation with the parameter combination "$k = 5, \sigma = 7.5$", our new implementation is roughly 744, 980 and 433 times faster than SW-PSSM for the three training data sets and 30, 8 and 9 times faster when classifying a single target. Additionally, in contrast to the profile kernel, simultaneously applying the model to multiple queries gains no speed-up. The runtime, therefore, is directly proportional to the number of queries and the superiority for predictions easily exceeds that of matrix creations for many queries. In summary, our implementation of the original profile kernel hugely outperforms SW-PSSM.

References

[1] Rui Kuang, Eugene Ie, Ke Wang, Kai Wang, Mahira Siddiqi, Yoav Freund, and Christina Leslie. Profile-Based String Kernels for Remote Homology

Detection and Motif Extraction. In *Proceedings of the 2004 IEEE Computational Systems Bioinformatics Conference*, CSB '04, pages 152–160, 2004.

[2] P.D. Sulatycke and K. Ghose. Caching-Efficient Multithreaded Fast Multiplication of Sparse Matrices. In *Parallel Processing Symposium, 1998. IPPS/SPDP 1998. Proceedings of the First Merged International ... and Symposium on Parallel and Distributed Processing 1998*, pages 117–123, 1998.

[3] Jean loup Gailly and Mark Adler. zlib, 2012. http://zlib.net.

[4] P. Deutsch. Deflate Compressed Data Format Specification version 1.3, 1996.

[5] Gaël Guennebaud, Benoît Jacob, et al. Eigen v3. http://eigen.tuxfamily.org, 2010.

[6] John C. Platt. Sequential Minimal Optimization: A Fast Algorithm for Training Support Vector Machines. Technical report, ADVANCES IN KERNEL METHODS - SUPPORT VECTOR LEARNING, 1998.

[7] T. Goldberg, T. Hamp, and B. Rost. LocTree2 Predicts Localization for All Domains of Life. *Bioinformatics*, 28(18):i458–i465, 2012.

[8] M. Schneider, L. Lane, E. Boutet, D. Lieberherr, M. Tognolli, L. Bougueleret, and A. Bairoch. The UniProtKB/Swiss-Prot Knowledgebase and its Plant Proteome Annotation Program. *J Proteomics*, 72(3):567–73, 2009.

[9] L. Fu, B. Niu, Z. Zhu, S. Wu, and W. Li. CD-HIT: Accelerated for Clustering the Next-generation Sequencing Data. *Bioinformatics*, 28(23):3150–2, 2012.

[10] John Fox. *Applied Regression Analysis, Linear Models, and Related Methods*. SAGE Publications, February 1997.

[11] B. Rost, G. Yachdav, and J. Liu. The PredictProtein Server. *Nucleic Acids Res*, 32(Web Server issue):W321–6, 2004.

[12] S. F. Altschul, T. L. Madden, A. A. Schaffer, J. Zhang, Z. Zhang, W. Miller, and D. J. Lipman. Gapped BLAST and PSI-BLAST: A New Generation of Protein Database Search Programs. *Nucleic Acids Res*, 25(17):3389–402, 1997.

[13] M. Magrane and U. Consortium. UniProt Knowledgebase: A Hub of Integrated Protein Data. *Database (Oxford)*, 2011:bar009, 2011.

[14] R. D. Finn, J. Clements, and S. R. Eddy. HMMER Web Server: Interactive Sequence Similarity Searching. *Nucleic Acids Res*, 39(Web Server issue):W29–37, 2011.

[15] M. Remmert, A. Biegert, A. Hauser, and J. Soding. HHblits: Lightning-fast Iterative Protein Sequence Searching by HMM-HMM Alignment. *Nat Methods*, 9(2):173–5, 2012.

[16] H. Rangwala and G. Karypis. Profile-based Direct Kernels for Remote Homology Detection and Fold Recognition. *Bioinformatics*, 21(23):4239–47, 2005.

[17] Christopher J. C. Burges. A Tutorial on Support Vector Machines for Pattern Recognition. *Data Min. Knowl. Discov.*, 2(2):121–167, 1998.
